# Changes in Fatty Acid Content in *Solanum* spp. Fruits during Ripening

**DOI:** 10.3390/plants12020268

**Published:** 2023-01-06

**Authors:** Jūratė Staveckienė, Jurgita Kulaitienė, Dovilė Levickienė, Nijolė Vaitkevičienė

**Affiliations:** Department of Plant Biology and Food Sciences, Vytautas Magnus University Agriculture Academy, Donelaičio Str. 58, 44248 Kaunas, Lithuania

**Keywords:** *Solanum* spp. fruits, ripening stage, species, saturated fatty acids, monounsaturated fatty acids, polyunsaturated fatty acids

## Abstract

This study aimed to determine the effects of the ripening stage and species on the contents of saturated (SFA), monounsaturated (MUFA), and polyunsaturated (PUFA) fatty acids in *Solanum* spp. fruits. A two-factor field experiment with four different *Solanum* spp. species (*S. nigrum*, *S. melanocerasum*, *S. retroflexum*, and *S. villosum*) and three ripening stages was conducted over two growing seasons (2020–2021). The fatty acid composition of the *Solanum* fruits was characterized using gas chromatography with a flame ionization detection. The results show that PUFAs are the dominant type of fatty acid in *Solanum* fruits, followed by MUFAs and SFAs. Overall, the highest PUFA contents were observed in *S. nigrum* fruits in the ripening stage I, and the highest MUFA and SFA contents were observed in *S. melanocerasum* fruits during ripening stages I and II, respectively.

## 1. Introduction

The genus *Solanum* belongs to the *Solanaceae* family, which are also known as wonderberry or sunberry [1]. The biochemical composition of *Solanum* spp. fruits is a new and promising research subject in European and Lithuanian agriculture sectors. Other sources report that this fruit has antibacterial, antidysenteric, and diuretic properties. They also contain naturally occurring substances with a pharmacological relevance, including steroidal lactones, glycosides, alkaloids, and flavonoids [2]. In their study of *S. retroflexum* fruits, D.V. Akishin et al. [3] revealed that fresh, fully ripe *Solanum* fruits are a valuable raw material for the production of healthy food because they contain high concentrations of a large number of biologically active compounds, such as 48.20 mg 100 g^−1^ ascorbic acid and 887.70 mg 100 g^−1^ anthocyanins. Moreover, fresh *S. nigrum* fruit have high antioxidant activity (229.40 mg 100 g^−1^) and are rich in protein (2.60%) and aromatic compounds (aldehydes, 158.90 mg 100 g^−1^; aliphatic monocarboxylic acids, 138.43 mg 100 g^−1^).

Numerous studies have investigated how ripening impacts the chemical composition of various fruits. Understanding how fruits’ chemical compositions change as a result of ripening can help increase their overall quality and nutritional value [4].

Fruit ripening includes biochemical, physiological, and structural changes, such as the production of secondary metabolites that affect the taste, fragrance, texture, and appearance [5]. As ripening occurs, these characteristics provide the first impression of a fruit’s quality. Pigments, such as lycopene and β-carotene, provide a visual indication that a fruit is mature and suitable for consumption [6].

Fatty acids are essential components of cellular membranes and storage lipids and are precursors for numerous plant metabolites, such as signaling molecules and phytoalexins [7,8]. According to the literature, oleic, linoleic, linolenic, palmitic, and stearic fatty acids are the most prevalent types of fatty acids in berries [9,10]. Monounsaturated fatty acids are known to lower the levels of “bad” cholesterol and to support the preservation of cell elasticity [11]. Polyunsaturated fatty acids are important for vital activity. They participate in the synthesis of important acids in the body, the lack of which leads to undesirable changes in the heart, kidneys, and reproductive organs [12]. Linoleic acid is the most useful and widely used polyunsaturated fatty acid in the pharmaceutical industry. As with all fatty acids, linoleic acid can be used as an energy source. It may be esterified to produce neutral and polar lipids, such as triacylglycerols, phospholipids, and cholesterol esters. Therefore, its content in food is significant [13].

There are few studies in the literature regarding the fatty acid content in *Solanum* fruits. The concentration of each bioactive compound changes according to the ripening stage and harvest time. It has been established that a series of biochemical reactions that lead to the production of various compounds occur during the ripening of *Solanum* spp. fruit [14].

Understanding the physicochemical characteristics of fruits during ripening is essential toward increasing the quantities of fatty acid compounds via species selection. Therefore, the aim of this study was to determine the effect of ripening stages on the quantities and qualities of fatty acids of the fruits of four *Solanum* spp.: *S. retroflexum*, *S. melanocerasum*, *S. nigrum*, and *S. villosum*.

## 2. Results and Discussion

### 2.1. Saturated Fatty Acids

**Figure 1:**
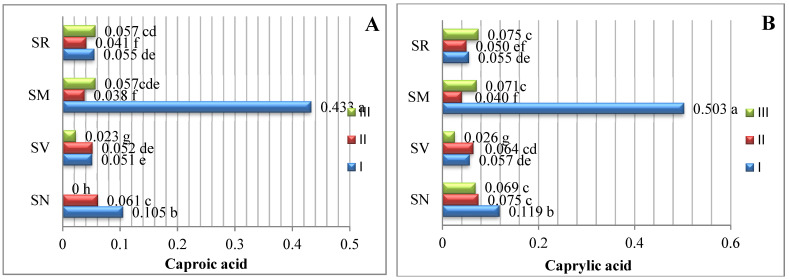

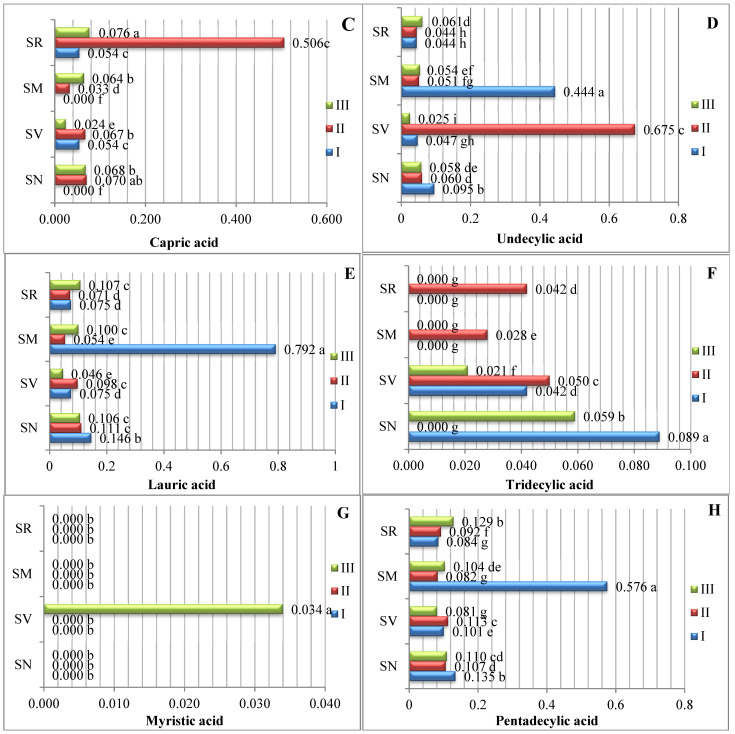

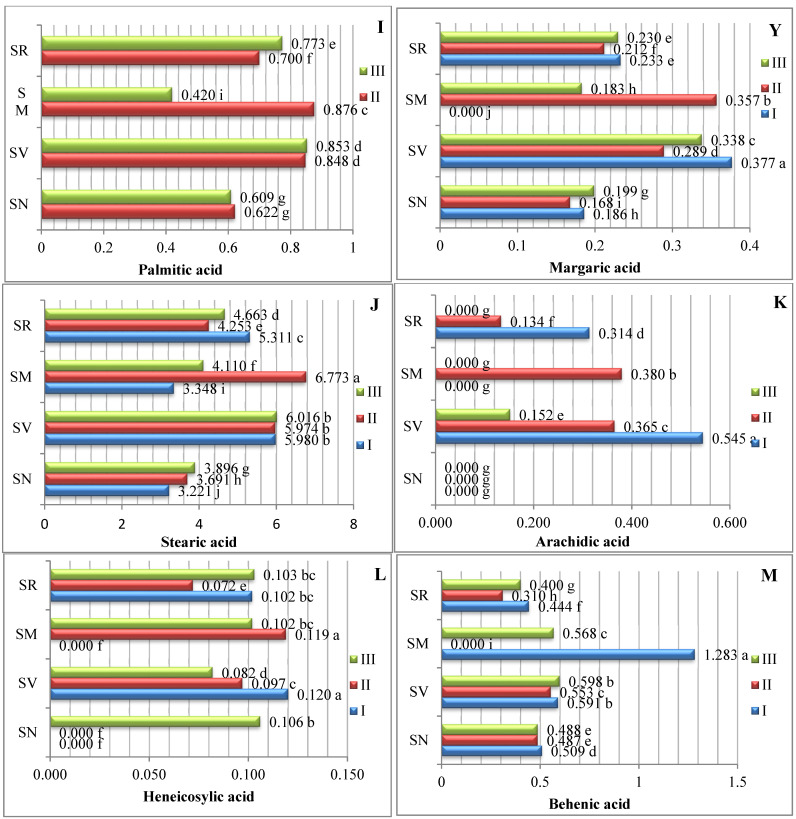

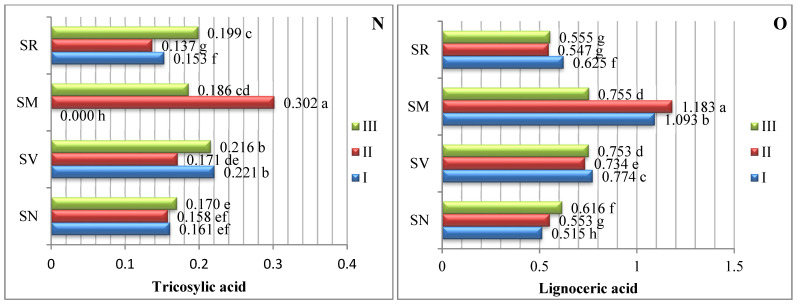
Amounts of saturated fatty acids in *Solanum* spp. fruit, %, in different ripening stages. Note: Percentages followed by different letters are statistically different (*p* < 0.05); *S. retroflexum*–(SR), *S. melanocerasum–*(SM), *S. nigrum*–(SN), *S. villosum*–(SV); ripening stages I, II, III.

The data averaged from two experimental years demonstrate that the amount of saturated fatty acids (SFAs) in *Solanum* spp. fruit varied according to the species and ripening stage (Figure 1). As there are insufficient studies describing variations in the fatty acid content of *Solanum* spp. fruits, we compared our data with those of other representatives of the *Solanum* genus. The analysis of the SFAs in unripe and ripened tomatoes showed variations in tridecylic acid (0.51–2.03%), myristic acid (0.36–0.29%), palmitic acid (16.6–17.9%), and stearic acid (4.87–6.24%) [15].

In our study, sixteen saturated fatty acids were identified in *S. retroflexum*, *S. nigrum*, *S. melanocerasum*, and *S. villosum* fruits (Figure 1); stearic acid was found to be the primary SFA, with levels ranging from 3.22 to 5.98%. J. L. Guil-Guerrero et al. [16] analyzed eight tomato cultivars, including “Cherry”, “Cherry Perra”, “Daniela Larga Vida”, “Lido”, “Pera Racimo”, “Raf”, and “Rambo” and concluded that stearic acid (3.22–5.19%) is not the most prevalent fatty acid. Our results show that palmitic acid (0.42–1.23%) is the second most predominant saturated fatty acid in the fruit from the four studied Solanum species. Compared with other ripening stages and species, the level of palmitic acid was considerably higher (1.23%) in the *S. melanocerasum* fruit harvested during stage I.

The analysis of our results indicates that the ripening stage and species do not influence variations in the caproic, caprylic, capric, undecylic, lauric, and margaric acids. Spyridon et al. 2020 [17] identified lower amounts of these acids in their research. Depending on the species, the contents of caproic acid varied from 0.028 to 0.073%, caprylic acid from 0.021 to 0.060%, and capric acid from 0.016 to 0.043%. A myristic acid content (0.034%) was found to be established in *S. villosum* fruits in ripening stage III, but this was not detected in *S. nigrum*, *S. retroflexum*, or *S. melanocerasum* fruits.

Villa-Rodrguez et al. [18] studied variations in the SFA content of “Hass” avocados and observed a significant increase in the total content during the fruit’s ripening. We also found that the pattern of fatty acid tends to vary based on the species and acid type.

### 2.2. Monounsaturated Fatty Acids

**Figure 2:**
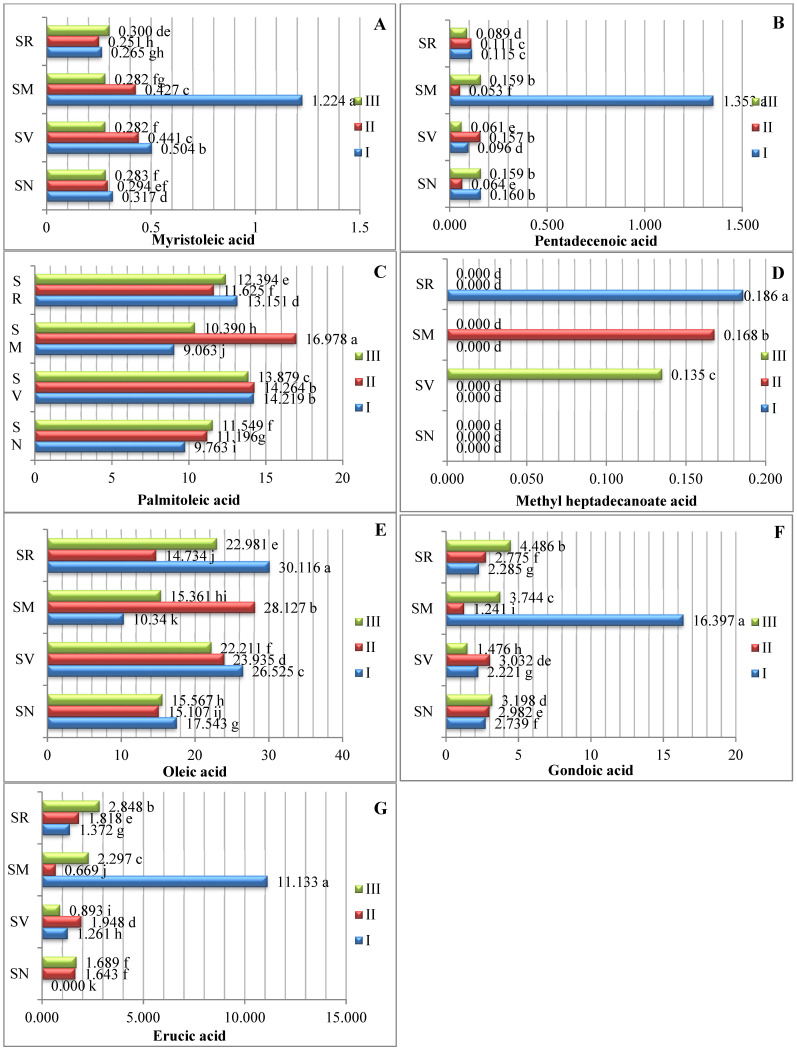
Amounts of monounsaturated fatty acids in *Solanum* spp. fruit, %, in different ripening stages. Note: Percentages followed by different letters are statistically different (*p* < 0.05); *S. retroflexum*–(SR), *S. melanocerasum*–(SM), *S. nigrum*–(SN), *S. villosum*–(SV); ripening stages I, II, III.

Seven monounsaturated fatty acids (MUFAs) were identified (Figure 2). Our data show that oleic acid was the predominant type of MUFA. The oleic acid contents ranged between 10.340% and 30.116%. Differences in the ripening stage and fruit species significantly influenced the oleic acid content, with the highest amount of oleic acid being found in *S. retroflexum* fruits in ripening stage I (30.116%). Palmitoleic acid was the second most predominant monounsaturated fatty acid; the analysis showed that the samples’ palmitoleic acid content ranged from 9.763% to 16.978%, with a significantly higher amount being found in the *S. melanocerasum* fruits in ripening stage II (16.978%). The study by Ramesh et al. in 2017 [15] showed that the oleic acid content was highest in the middle ripening stage (when tomatoes were not fully ripe), at 26.6%; it was 23.6% in the first ripening stage and 20.6% in the fully ripened stage.

Our results show that the myristoleic acid and pentadecanoic acid contents ranged from 0.251% to 1.224% and 0.064% to 1.354%, respectively (Figure 2). We determined that the *S. melanocerasum* species had significantly higher levels of both acids in ripening stage I. In their study of tomato seeds, A. Demirbas [19] found 0.2% myristoleic acid. During the ripening period, the *S. melanocerasum* species had a significantly higher contents of gondoic (16.397%) and erucic (11.133%) acids in ripening stage I.

Compared with other *Solanum* fruits in our study, the *S. retroflexum* fruits harvested in ripening stage I showed significantly higher amounts of elaidic acid (11.561%). In *S. melanocerasum* fruit, the content of elaidic acid (10.222%) peaked during ripening stage II.

Our results show that methyl heptadecanoate acid was present in lower concentrations, varying from 0.135% to 0.186%, and was detected only in *S. retroflexum* fruits in ripening stage I, in *S. melanocerasum* fruits in stage II, and in *S. villosum* fruits in stage III.

### 2.3. Polyunsaturated Fatty Acids

**Figure 3:**
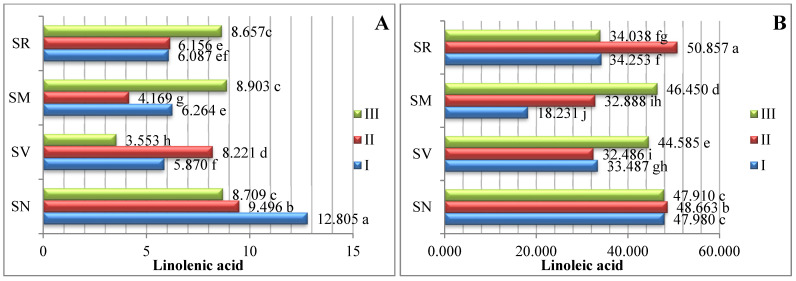

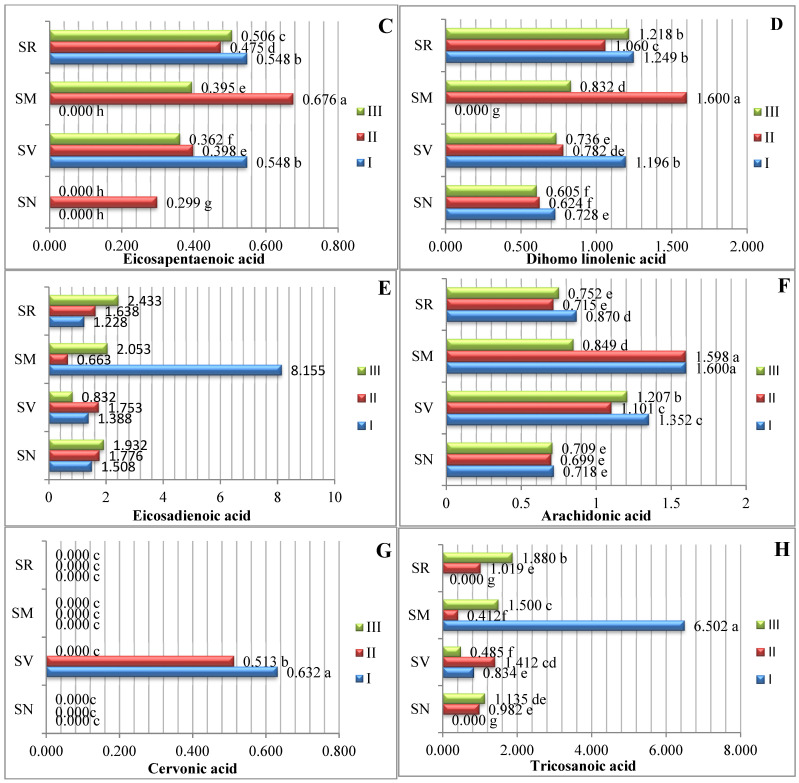
Amounts of polyunsaturated fatty acids in *Solanum* spp. fruit, %, in different ripening stages. Note: Percentages followed by different letters are statistically different (*p* < 0.05); *S. retroflexum*–(SR), *S. melanocerasum*–(SM), *S. nigrum*–(SN), *S. villosum*–(SV); ripening stages I, II, III.

The studied fruits were found to contain eight different types of PUFAs (Figure 3). The predominant PUFA in the *Solanum* spp. fruit was linoleic acid, which varied between 18.231% and 50.857%. The *S. retroflexum* samples contained a significantly higher amount (50.875%) of linoleic acid in ripening stage II. Other researchers have established that linoleic acid ranges between 47.80% and 53.44% as the predominant PUFA in tomato fruit (*Solanum lycopersicum* L.) [17].

Linolenic acid was the second most predominant polyunsaturated fatty acid; the linolenic acid amounts varied from 3.553% to 12.805% in the analyzed fruits harvested three times throughout the growing season. The *S. nigrum* samples harvested in ripening stage I had the highest linolenic acid content (12.805%). A similar amount of linolenic fatty acid was found in the study of Spyridon et al. [17], where the content of linolenic acid in tomato fruit during the ripening stages was established to range from 5.52% to 8.02%. In their research, J. Kulaitienė et al. [20] determined the effect of ripening stages on the quality and quantity of fatty acids in the seeds of two rosehip species and two cultivars. Linolenic acid was found to be the second most abundant fatty acid, with contents ranging from 19.305% to 30.645% at the full ripening stage.

*S. melanocerasum* fruits in ripening stage I had significantly higher amounts of eicosadienoic (8.155%) and n-tricosanoic (6.502%) acids. During the ripening period, the amount of eicosadienoic and n-tricosanoic acids significantly decreased by 8.14 and 3.4 times, respectively.

Linoleic, linolenic, and arachidonic acids show starkly different patterns of accumulation during the vegetation period.

In assessing the profiles of all the various PUFAs, cervonic acid was found in the lowest amounts; it was found only in *S. villosum* fruits in ripening stages I and II, at 0.632% and 0.513%, respectively. However, there are no research data regarding fatty acids, such as cervonic acid, in Solanum fruits. In their investigation, Pieszka et al. [21] found that black currant seeds contain 0.01% cervonic acid.

### 2.4. Total Amounts of SFAs, MUFAs, and PUFAs

**Figure 4:**
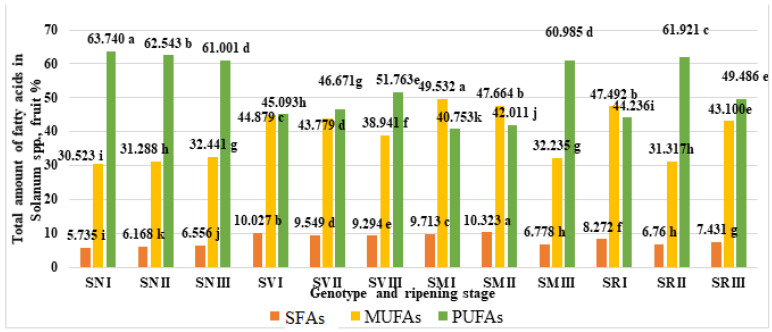
Total amounts of saturated (SFA), monounsaturated (MUFA), and polyunsaturated (PUFA) fatty acids in *Solanum* spp. fruit, %, in different ripening stages. Note: Percentages followed by different letters are statistically different (*p* < 0.05); *S. retroflexum*–(SR), *S. melanocerasum*–(SM), *S. nigrum*–(SN), *S. villosum*–(SV); ripening stages I, II, III.

According to our data, PUFAs were the primary fatty acids (Figure 4). The results show that the highest PUFA content (63.74%) was found for *S. nigrum* fruit in ripening stage I, whereas the lowest PUFA content (40.753%) was observed in *S. melanocerasum*. fruit, also in ripening stage I.

During ripening stages I and II, *S. melanocerasum* fruits contained the highest amounts of MUFAs (49.532%) and SFAs (10.323%), respectively. Our findings indicate that the MUFA, SFA, and PUFA contents in the fruits of the same species vary significantly throughout their ripening stages.

## 3. Materials and Methods

### 3.1. Field Experiment

**Figure 5:**
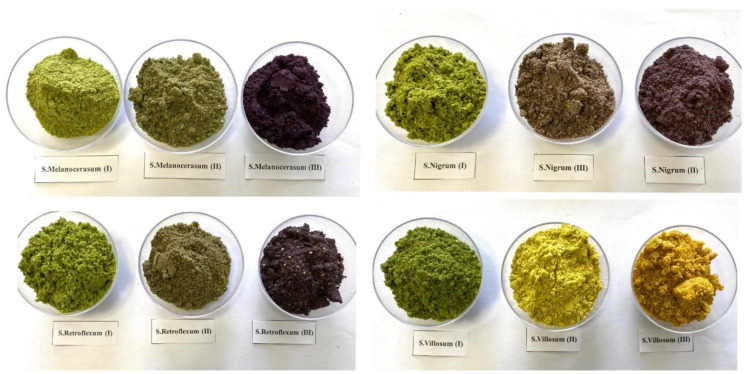
Fruit ripening stages of *Solanum* spp. represented by milled fruit samples. (photos by J. Staveckienė).

The two-factor experiment was conducted in 2020–2021 on Mariaus Stavecko farm in the Kaunas district, Lithuania (WGS coordinates 54.8719020, 23.8672686). Factor A is the fruit of four *Solanum* spp. species: *S. retroflexum*, *S. melanocerasum*, *S. nigrum*, and *S. villosum*. Factor B is three fruit ripening stages: stage I, fruit color green (30% maturity); stage II, fruit color 40–60% purplish-violet or yellow-orange (60% maturity), inside incompletely ripe; and stage III, fruit color 100% velvety black-blue or orange, inside fully ripe (100% maturity) (Figure 5) [22,23].

In the field experiment, the seedlings were planted in the soil. The seeds were sown in nurseries in March, and the strongest seedlings were transferred to the field in the 3rd ten-day period of May. Before planting, the soil surface was covered with a black agro film; holes were cut in the film, and the seedlings were then planted in these holes. A drip irrigation system was installed under the agro film; the watering rate was 1 L per hour, as needed, considering the meteorological conditions.

Experimental plots were arranged in randomized blocks with four replicates for each treatment. Each replication consisted of four seedlings of each species. Each experimental plot was 7.5 m long and 1.5 m wide. The entire experimental area was 148 m^2^, including the protective zone.

Fruits were randomly collected for analysis from July 1st through to the first frost, according to the ripening stage. In each experimental year, the harvest dates depended on the meteorological conditions and the fruit ripening stages, which were visually assessed. For the laboratory analyses, 30–40 fruit samples were randomly harvested from each block of each treatment for preparing a 1.5 kg composite fruit sample.

### 3.2. Preparation of Samples

The fruits were washed using tap water, dried, and stored at −34 °C. The samples were lyophilized for 24 h using a Freeze–Drying Plant Sublimator 3′4′5 (ZIRBUS Technology GmbH, Bad Grund, Germany). After lyophilization, the fruits were milled (Grindomix GM 200, Retsch GmbH, Haan, Germany) and stored in airtight containers at 5 °C in the dark until the chemical analysis.

### 3.3. Soil Agrochemical Analyses

Soil samples were taken in spring from the arable layer (0–20 cm depth) using an agrochemical auger. The soil samples were air-dried in open plastic boxes, homogenized, and sieved through a 1 mm mesh sieve. The agrochemical analyses of the experimental soil were conducted at the Laboratory of Analyses of Vytautas Magnus University Agriculture Academy. The soil samples were analyzed for the pH, KCl, and phosphorus, potassium, and total nitrogen contents. The soil’s pH was measured according to the potentiometric method using a pH-meter in 1 N of KCl extract [24]. The available phosphorus and potassium were extracted using ammonium–lactate according to the Egner–Riehm–Domingo method [25]. The total nitrogen concentration (mg kg^−1^) was determined using the Kjeldahl method.

The experimental field soil was characterized by the acidity (pH = 4.16), medium potassium status (K_2_O = 78.5–102.2 mg kg^−1^), low phosphorus status (P_2_O_5_ = 45.9–69.3 mg kg^−1^), and 1.25% total nitrogen content.

### 3.4. Determination of Fatty Acid Content

The fatty acid contents of the lyophilized fruit powders were determined via gas chromatography with flame ionization detection. The test samples were prepared in accordance with LST EN ISO 12966-2:2011 for the analysis of the fatty acids. The fatty acids were methylated using anhydrous KOH in methanol. The chromatographic analysis of the methyl esters of fatty acids was performed using a gas chromatograph Shimadzu GC-2010 and a BPX-70 120 m column in accordance with LST EN IS 15304:2003/ac:20052. The following quantitative ratios of 31 fatty acids were estimated in the fruits: C6:0 (caproic acid), C8:0 (caprylic acid), C10:0 (capric acid), C11:0 (undecylic acid), C12:0 (lauric acid), C13:0 (tridecylic acid), C14:0 (myristic acid), C15:0 (pentadecylic acid), C16:0 (palmitic acid), C17:0 (margaric acid), C18:0 (stearic acid), C20:0 (arachidic acid), C21:0 (heneicosylic acid), C22:0 (behenic acid), C23:0 (tricosylic acid), C24:0 (lignoceric acid), C14:1 (myristoleic acid), C15:1 (pentadecenoic acid), C16:1 (palmitoleic acid), C17:1 (methyl heptadecanoate acid), C18:1 (oleic acid), C20:1 (gondoic acid), C22:1 (erucic acid), C18:3 (linolenic acid), C18:2 (linoleic acid), C20:5 (eicosapentaenoic acid), C20:3 (dihomo linolenic acid), C20:2 (eicosadienoic acid), C20:4 (arachidonic acid), C22:6 (cervonic acid), and C23:2 (tricosanoic acid).

The analysis conditions were as follows. Column temperature: 60 °C for 2 min, 200 °C/min up to 230 °C, holding for 45 min, evaporator temperature 250 °C, flame ionization detector temperature 270 °C, gas carriers, and nitrogen. Fatty acid set “supelco 37 component” was used to identify the fatty acids in the “FAME mix”. The tetradecadiene and hexadecadiene fatty acids were identified via interpolation.

The preparation of the samples was as follows: 15 ± 0.01 g of each sample was weighed, poured into 25 mL of chromatographically clean hexane, and then the fat was extracted by stirring for 1 h. Next, 4 mL of the extract was poured into a test tube, 200 µL of 2 mol/L KOH solution was added, and the mixture was then centrifuged and left for 30 min for exfoliation. A 2 mL aliquot was taken from each prepared sample and poured into the bottle of the automatic feeding system. An automatic syringe removed 1 µL, which was injected into the chromatographic evaporator. A chromatographic analysis was performed.

### 3.5. Statistical Analysis

The data for the *Solanum* spp. fatty acid contents were processed using Microsoft^®®^ Excel^®®^ 2016 MSO software and the STATISTICA 10 (StatSoft, Inc., Tulsa, OK, USA, 2010) package. The reliability of the results was evaluated using a two-way analysis of variance (ANOVA software package). The statistical significance of the differences between the means was estimated using Fisher’s LSD test (*p* < 0.05).

## 4. Conclusions

Our results demonstrate that the quality and quantity of the fatty acids in all of the investigated fruits is dependent on the species and ripening stage. The polyunsaturated fatty acids were the most prevalent type, with the contents ranging from 40.753% to 63.740% in all of the investigated species during their ripening. Linoleic acid was the most abundant polyunsaturated fatty acid. Significantly, the highest total amount (49.532%) of monounsaturated fatty acids was identified in *S. melanocerasum* fruits in ripening stage I, and oleic acids were the most abundant among monounsaturated fatty acids. The highest amount of saturated fatty acids (10.323%) of all the tested samples was observed for *S. melanocerasum* fruits during ripening stage II. Stearic acid and palmitic acid were the two predominant SFAs.

## Data Availability

Not applicable.

## References

[1] Knapp S., Bohs L., Nee M., Snpooner D.M. (2004). Solanaceae–A model for linking genomics with biodiversity. Comp. Funct. Genom..

[2] Kumar P., Kumar J., Kumar R., Dubey R.C. (2016). Studies on phytochemical constituents and antimicrobial activities of leaves, fruits and stems of *Solanum nigrum* L.. Asian J. Plant. Sci. Res..

[3] Akishin D.V., Vinnitskaya V.F., Vetrov M.Y. (2017). Functional and nutritional value of fresh and processed fruit nightshade Sunberry. Technol. Food Process. Ind. AIC–Health Food.

[4] Tang Z.X., Shi L.E., Aleid S.M. (2013). Date fruit: Chemical composition, nutritional and medicinal values, products. J. Sci. Food Agric..

[5] Konozy E.H.E., Causse M., Faurobert M. (2012). Cell wall glycosidase activities and protein content variations during fruit development and ripening in three texture contrasted tomato cultivars. Saudi J. Biol. Sci..

[6] Klee H.J., Giovannoni J.J. (2011). Genetics and control of tomato fruit ripening and quality attributes. Ann. Rev. Genet..

[7] Ohlrogge J., Browse J. (1995). Lipid biosynthesis. Plant Cell.

[8] Lim G.H., Singhal R., Kachroo A., Kachroo P. (2017). Fatty acid- and lipidmediated signaling in plant defense. Ann. Rev. Phytopathol..

[9] Johansson A., Laakso P., Kallio H. (1997). Characterization of seed oils of wild, edible Finnish berries. Z. Lebensm. Unters. Forsch. A.

[10] Yang B., Koponen J., Tahvonen R., Kallio H. (2003). Plant sterols in seeds of two species of Vaccinium (*V. myrtillus* and *V. vitis-idaea*) naturally distributed in Finland. Eur. Food Res. Technol..

[11] Mensink R.P., Katan M.B. (1989). Effect of a diet enriched with monounsaturated or polyunsaturated fatty acids on levels of low-density and high-density lipoprotein cholesterol in healthy women and men. N. Engl. J. Med..

[12] Trautwein E.A. (1998). L‘huile de colza: Un produit de haute valeur pour l‘alimentation humaine. Rev. Suisse Agr..

[13] Jay W., Kevin F. (2013). Linoleic Acid. Adv. Nutr..

[14] Budryn G., Nebesny E. (2006). Fenolokwasy–Ich właściwości, występowanie w surowcach roś-linnych, wchłanianie i przemiany metaboliczne. Bromatol. Chem. Toksykol..

[15] Ramesh K.S., Ahmad J.Z., Young-Soo K. (2017). Ripening Improves the Content of Carotenoid, α-Tocopherol, and Polyunsaturated Fatty Acids in Tomato (Solanum lycopersicum L.) Fruits.

[16] Guil-Guerrero J.L., Rebolloso-Fuentes M.M. (2009). Nutrient composition and antioxidant activity of eight tomato (*Lycopersicon esculentum*) varieties. J. Food Compost. Anal..

[17] Spyridon A., Petropoulos S.A., Fernandes Â., Xyrafis E., Polyzos N., Antoniadis V., Barros L.C.F.R., Ferreira I. (2020). The optimization of nitrogen fertilization regulates crop performance and quality of processing tomato (*Solanum lycopersicum* L. cv. Heinz 3402). Agronomy.

[18] Villa-Rodríguez J.A., Molina-Corral F.J., Ayala-Zavala J.F., Olivas G.I., González-Aguilar G.A. (2011). Effect of maturity stage on the content of fatty acids and antioxidant activity of “Hass” avocado. Food Res. Int..

[19] Demirbas A. (2010). Oil, micronutrient and heavy metal contents of tomatoes. Food Chem..

[20] Kulaitienė J., Medveckienė B., Levickienė D., Vaitkevičienė N., Makarevičienė V., Jarienė E. (2020). Changes in fatty acids content in organic rosehip (*Rosa* spp.) seeds during ripening. Plants.

[21] Pieszka M., Migdał W., Gąsior R., Rudzińska M., Bederska-Łojewska D., Pieszka M., Szczurek P. (2015). Native Oils from Apple, Blackcurrant, Raspberry, and Strawberry Seeds as a Source of Polyenoic Fatty Acids, Tocochromanols, and Phytosterols: A Health Implication. J. Chem..

[22] Park M.H., Sangwanangkul P., Baek D.R. (2018). Changes in carotenoid and chlorophyll content of black tomatoes (*Lycopersicone sculentum* L.) during storage at various temperatures. Saudi J. Biol. Sci..

[23] Coyago-Cruz E., Corell M., Moriana A., Hernanz D., Stinco C.M., Mapelli-Brahm P., Meléndez-Martínez A.J. (2021). Effect of regulated deficit irrigation on commercial quality parameters, carotenoids, phenolics and sugars of the black cherry tomato (*Solanum lycopersicum* L.) ‘Sunchocola’. J. Food Compos. Anal..

[24] (2005). Soil Quality. Determination of pH.

[25] Oreshkin N. (1980). Extraction of mobile forms of phosphorus and potassium by the Egner–Riehm–Domingo method. Agrokhimiia.

